# Evaluation of the *in vitro* trypanocidal activity of methylated flavonoid constituents of *Vitex simplicifolia* leaves

**DOI:** 10.1186/s12906-015-0562-2

**Published:** 2015-03-26

**Authors:** Ngozi Nwodo, Festus Okoye, Daowan Lai, Abdesammed Debbab, Marcel Kaiser, Reto Brun, Peter Proksch

**Affiliations:** Department of Pharmaceutical and Medicinal Chemistry, University of Nigeria, Nsukka, Nigeria; Department of Medical Parasitology and Infection Biology, Swiss Tropical and Public Health Institute, CH-4002 Basel, Switzerland; Institute for Pharmaceutical Biology and Biotechnology, Heinrich-Heine-University, D- 40225 Dusseldorf, Germany; Department of Pharmaceutical Chemistry, Nnamdi Azikiwe University, Awka, Anambra State Nigeria; University of Basel, CH-4003 Basel, Switzerland

**Keywords:** *Vitex simplicifolia*, Verbenaceae, Flavonoids, *Trypanosoma brucei rhodesiense*

## Abstract

**Background:**

Trypanosomiasis is a neglected tropical disease with complex clinical manifestations, tedious diagnosis, and difficult treatments. The drugs available for the treatment of this endemic disease are old, expensive, and associated with other problems including safety and drug resistant parasites. Therefore, there is an urgent need for the development of new, effective, cheap, and safe drugs for its treatment. Plants are potentially rich sources of leads for new drugs against trypanosomiasis.

*Vitex simplicifolia* (Verbenaceae) is used traditionally for the treatment of tooth ache, edema, skin diseases, gout and trypanosomiasis in Nigeria. In a preliminary study, the methanol extract of *Vitex simplicifolia* was shown to exhibit a pronounced trypanocidal activity against *T. b. rhodesiense.*

The present study was undertaken to investigate the active component responsible for the acclaimed activity of the leaves of *Vitex simplicifolia* in the traditional treatment of trypanosomiasis in Nigeria and other African countries. Our investigations aim at assessing the plant as a new source of potential trypanocidal compounds.

**Methods:**

The crude extracts were prepared from the dried leaves using methanol, successive extraction with hexane, dichloromethane, ethylacetate and butanol was also done. The ethylacetate fraction was further fractionated and compounds isolated using preparative chromatographic technique and their structures were elucidated by NMR, mass spectrometry and comparison with literature data. Trypanocidal activities and cytotoxicity, using rat skeletal myoblast (L6) cells were investigated and their selectivity indices were determined.

**Results:**

The chromatographic separations of the methanol extracts gave rise to seven compounds. The isolated compounds **2**, **3**, **6** and **7** exhibited promising trypanocidal activity with IC_50_ values ranging from 4.7-12.3 μg/ml and cytotoxicity in the range of 1.58- 46.20 μg/ml. Compound **6,** however, showed the most selective trypanocidal activity with a selectivity index of 9.8. This is the first report of trypanocidal activity of flavonoids from this plant genus.

**Conclusions:**

The isolated compounds from *Vitex simplicifolia* exhibited noteworthy trypanocidal activities and hence may provide a source of new antitrypanosomal agents. These results also support the traditional use of *Vitex simplicifolia* in the treatment of trypanosomiasis. This is the first report of trypanocidal effect of flavonoids from this plant genus.

## Background

Human African trypanosomiasis also known as African sleeping sickness is caused by the protozoan parasites *Trypanosoma brucei rhodesiense* and *Trypanosoma brucei gambiense* in countries of sub-Saharan Africa [1]. World Health Organization (WHO) categorized it among the neglected tropical diseases which threatens primarily rural populations and is fatal unless treated. The use of the currently available drugs for the treatment of sleeping sickness has been limited owing to their numerous side effects, difficulty in administration and a certain loss of efficacy [2]. There is a great need for new safe chemotherapeutic agents preferably with oral application. In the drug pipeline managed by the Drugs for Neglected Diseases initiative (DNDi) two molecules are in clinical trial phases, fexinidazole and SCYX-7158 [3,4]. Drug discovery efforts are also directed towards natural products as an alternative source to synthetic compounds. Studies have revealed that many plant species are potential sources of novel trypanocidal compounds [5-9].

*Vitex simplicifolia* (Verbenaceae) is a sprawling shrub that can grow as tall as 1.5 m in height [10]. The leaves are strongly aromatic, intensifying when crushed and measure 2–6.5 cm in length and 1–4.5 cm in width [11]. The leaves and the edible fruits of *V. simplicifolia* are used in traditional medicine for the treatment of malaria, skin diseases, toothache, edema, gout and dermatitis [12]. In southern China, *V. simplicifolia* is used for the treatment of various pain disorders, such as stomach ache, hernia ache, dysmenorrhea, arthralgia, and piles [13]. Over the years, folk medicine has remained a veritable platform for researchers for sourcing lead compounds for the development of potent therapeutic agents. Several trypanocidal molecules have been isolated from plants with undefined side effects [14,15]. Flavonoids are one of the largest and most abundant classes of secondary metabolites in plants and mainly found in fruits and vegetables. Many flavonoids of plant origin have shown to possess a variety of medicinal properties [16-18]. Previous studies have demonstrated some structure activity relationships among flavonoid derivatives and their trypanocidal and /or antileishmanial activities [19-23]. So far, no similar flavonoids or its derivatives have been reported to possess trypanocidal activity against African trypanosomes. In this study, we report for the first time the trypanocidal activity of some methylated flavonoid derivatives from *Vitex simplicifolia*.

## Methods

### General experimental procedures

Optical rotations were determined on a Perkin-Elmer-241 MC polarimeter. 1D and 2D NMR spectra were recorded on an Avance DMX 600 NMR spectrometer. Chemical shifts were referenced to the residual solvent peak at δ_H_ 7.26 (CDCl_3_) and 2.05 (acetone-*d*_6_) for ^1^H, and δ_C_ 77.0 (CDCl_3_) and 29.92 (acetone-*d*_6_) for ^13^C, respectively. Mass spectra were measured with a LCMS HP1100 Agilent Finnigan LCQ Deca XP Thermoquest and high-resolution electrospray ionization mass spectroscopy (HRESIMS) were recorded with an UHR-TOF maXis 4G (Bruker Daltonics, Bremen) mass spectrometer. HPLC analysis was performed with a Dionex P580 system coupled to a photodiode array detector (UVD340S); routine detection was at 235, 254, 280, and 340 nm. The separation column (125 × 4 mm) was prefilled with Eurosphere-10 C18 (Knauer, Germany), and the following gradient was used (MeOH, 0.1% HCOOH in H_2_O): 0–5 min (10% MeOH); 5–35 min (10-100% MeOH); 35–45 min (100% MeOH).

Semi-preparative HPLC was performed using a Merck Hitachi HPLC System (UV detector L-7400; Pump L-7100; Eurosphere-100 C18, 300 × 8 mm, Knauer, Germany). Column chromatography was performed on Silica gel 60 M (230–3400 mesh ASTM, Macherey-Nagel GmbH & Co. KG, Dueren, Germany) and Sephadex LH-20 (Sigma). TLC was carried out on precoated silica gel plates (silica gel 60 F-254, Merck KGaA, Darmstadt, Germany) for monitoring of fractions. Detection was performed at 254 and 366 nm.

### Plant material

The leaves of *Vitex simplicifolia* were collected between March and April 2012 from Nsukka, Enugu State, Nigeria. The plant material was authenticated by Mr. Alfred Ozioko of the Centre for Ethnomedicine and Drugs Development, a subsidiary of Bioresources Development and Conservation Program (BCDP), Nsukka, Enugu State. The voucher specimens were deposited at the herbarium of the Department of Pharmacognosy, University of Nigeria, Nsukka. The leaves were cleaned, dried under room temperature and pulverized.

### Extraction and isolation procedures

About 600 g of the powdered leaves of *V. simplicifolia* was extracted with 2.5 l of methanol for 24 hours with constant stirring using a magnetic stirrer. The methanol extract (45 g) was dispersed in water and successively extracted with hexane, dichloromethane (DCM), ethylacetate and butanol. The chromatographic separations of the DCM fraction (428.8 mg), using Vacuum Liquid Chromatography (VLC) 22 x 4 cm on silica gel (230–400 mesh) with gradient of n-hexane: ethylacetate led to 10 VLC fractions A-J. Fraction E (72.2 mg) eluted with 50% n-hexane: ethylacetate was subjected to semi-preparative HPLC (Merck, Hitachi L-7100) using a Eurosphere 100–10 C18 column (300 × 8 mm, i.d.) with the following gradient (MeOH:H2O): 0 mins, 50% MeOH; 0 mins, 50% MeOH; 4 mins, 60% MeOH; 5 mins, 65% MeOH; 12 mins, 85% MeOH; 14 mins 90% MeOH; 15 mins, 100% MeOH; 16 mins, 100% MeOH to obtain compounds **1** (8.0 mg), **2** (5.8 mg), **3** (1.8 mg), **4** (1.9 mg), **5** (3.0 mg), **6** (1.8 mg) and **7** (2.0 mg).

### Determination of *in vitro* trypanocidal activity and cytotoxicity

Minimum essential medium (50 μl) supplemented according to a standard method [24], with 2-mercaptoethanol and 15% heat inactivated horse serum was added to each well of a 96-well microtiter plate. Serial three-fold compound dilutions were prepared covering a range from 90 to 0.123 μg/ml. Then 10^4^ bloodstream forms of *Trypanosoma brucei rhodesiense* STIB 900 (a clone of a population isolated in 1982 from a patient in Tanzania) in 50 μl culture medium were added to each well and the plate incubated at 37°C under a 5% CO_2_ atmosphere for 72 h. Ten microlitres of Alamar Blue (12.5 mg resazurin dissolved in 100 ml distilled water) were then added to each well and incubation continued for a further 2–4 h. The plate was then read in a Spectramax Gemini XS microplate fluorometer (Molecular Devices Cooperation, Sunnyvale, CA, USA) using an excitation wavelength of 536 nm and emission wavelength of 588 nm [25]. Fluorescence development was measured and expressed as percentage of the control. Data were transferred into the graphic programme Softmax Pro (Molecular Devices) which calculated IC_50_ values. Melarsoprol was used as standard drug.

Cytotoxicity was determined using a rat skeletal myoblast cell line (L6 cells). The culture medium was RPMI 1640 supplemented with L-glutamine 2 mM, HEPES 5.95 g/l, NaHCO_3_ 2 g/l and 10% foetal bovine serum. Podophyllotoxin (Sigma-Aldrich) was used as the reference drug. The assay was performed following the antitrypanosomal assay protocol as described above. The IC_50_ values were calculated from the sigmoidal growth inhibition curves using Softmax Pro software (Molecular Devices Corp.). Tests were done in three independent experiments in duplicate.

## Results and discussion

In a previous preliminary study, the methanol extract of *Vitex simplicifolia* was shown to exhibit a moderate trypanocidal activity against *T. b. rhodesiense* with an IC_50_ value of 14.2 μg/ml [26]. The methanol extract was therefore subjected to further fractionation and purification to identify the active components leading to the isolation of seven flavonoid derivatives (**1**–**7**).

**Figure 1:**
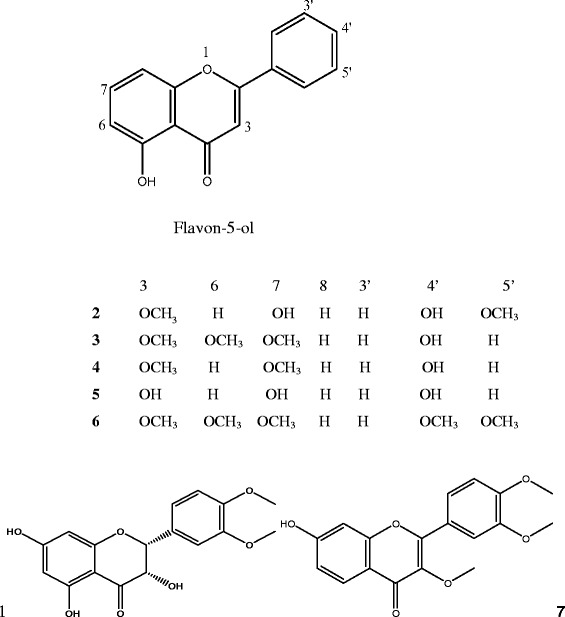
**Chemical structures of the seven flavonoid derivatives isolated from** ***Vitex simplicifolia*** **(**1**–**7**).**

The structures of the isolated flavonoid derivatives in Figure 1 were elucidated by a combination of 1D and 2D NMR and mass spectral analyses and comparison of the data with those reported in the literature as 2-(5′-methoxyphenyl)-3,4′,5,7,8-trihydroxychroman-4-one (**1**), 2-(5′-methoxyphenyl) 4′,5,7-trihydroxy-3-methoxychromen-4-one (**2**), Penduletin (**3**), 2-(4′-hydroxyphenyl)-5-hydroxy 3,7- dimethoxy chromen-4-one (**4**), 2-(4-hydroxyphenyl)-3,5,7-trihydroxy chromen-4-one (**5**), Artemetin (**6**) and 2-(3′,4′-dimethoxyphenyl)-7-hydroxychromen-4-one (**7**). All the seven compounds in Figure 1 were isolated from *Vitex simplicifolia* for the first time. Compounds **3** and **6** have been isolated from a related *Vitex* species *Vitex trifolia*, while the others were isolated from the genus *Vitex* for the first time.

All isolated compounds were subjected to *in vitro* assays assessing the trypanocidal and cytotoxic activities. The results are shown in Table 1.

**Table 1:** ***In vitro*** **trypanocidal activity and cytotoxicity of flavonoids from** ***Vitex simplicifolia*** **against** ***T.b rhodesiense*** **and L6 cells respectively**

**Compound**	***T.b.rhodesiense***	**L6 Cells**	**SI**
	**IC**_**50**_ **μg/ml**	**IC**_**50**_ **μg/ml**	
1	10.2	100	9.8
2	12.3	6.64	0.5
3	13.8	14.0	1.0
4	19.4	28.2	1.4
5	23.7	100	4.2
6	4.7	46.2	9.8
7	10.8	1.58	0.2
Melarsoprol	0.002		
Podophyllotoxin		0.005	

SI: selectivity index (IC_50_ of L6 cells/ IC_50_ of *T.b.rhodesiense*)*.*

Four of the isolated compounds **2**, **3**, **6** and **7** exhibited moderate trypanocidal activities with IC_50_ values ranging from 4.7-13.8 μg/ml, however, selectivity versus mammalian L6 cells was completely missing for 2, 3, and 7. Compound **6** showed the most promising and selective trypanocidal activity (IC_50_ = 4.7 μg/ml) with a selectivity index of 9.8. A close look at the structures of the isolated compounds reveals some structural features that can be correlated with the activities. The trypanocidal activity appears to increase with increase in the methylation of the hydroxyl groups. A plausible explanation for this observation is the corresponding increase in lipophilicity, which increases the permeability of the compounds across the parasite’s membranes. This is in line with other investigations which stated that substitution at C-4” especially with methoxyl group seems relevant for antiplasmodial activity [20]. Similar studies inferred that methylation at C-4” of the flavonols potentiates the trypanocidal activities [18]. Another report stated that association of methoxyl group at C-7 with hydroxyl group at C-4” is responsible for the trypanocidal activities of some flavonoids [27]. It might as well imply that hydroxyl at C-7 associates with the methoxyl group at C-4”. In this study, it can be inferred that the presence of methoxyl group at C-3, which is common to all the active compounds in this case, could be attributed to their activities. Furthermore, the absence of an OH group on the ring B which is the case with the most active compound could be another reason for its activity. According to a similar finding, the effect of the methoxyl groups on the flavonoid rings in relation to our findings needs further classifications [19].

## Conclusions

In conclusion, the flavonoids isolated from *V. simplicifolia* showed low to moderate trypanocidal activity *in vitro* with compound **6** as the most active of the isolated molecules with a promising trypanocidal activity and some selectivity. A similar study shows compounds isolated from plants with IC_50s_ from 1.6 to 19.4 μM and selectivity indices between 0.5 and 6.5 [14]. To the best of our knowledge this is the first report on trypanocidal activity of this class of compounds from this plant genus. Future optimization of these compounds through structural alteration may lead to molecules with improved trypanocidal activity and selectivity.
